# Magnetic capture from blood rescues molecular motor function in diagnostic nanodevices

**DOI:** 10.1186/1477-3155-11-14

**Published:** 2013-05-03

**Authors:** Saroj Kumar, Lasse ten Siethoff, Malin Persson, Nuria Albet-Torres, Alf Månsson

**Affiliations:** 1 School of Natural Sciences, Linnaeus University, Kalmar SE-391 82, Sweden

**Keywords:** Magnetic nanoparticle, Biomolecular motor, Myosin, Nanoseparation, Lab-on-a-chip, Bioconjugation

## Abstract

**Background:**

Introduction of effective point-of-care devices for use in medical diagnostics is part of strategies to combat accelerating health-care costs. Molecular motor driven nanodevices have unique potentials in this regard due to unprecedented level of miniaturization and independence of external pumps. However motor function has been found to be inhibited by body fluids.

**Results:**

We report here that a unique procedure, combining separation steps that rely on antibody-antigen interactions, magnetic forces applied to magnetic nanoparticles (MPs) and the specificity of the actomyosin bond, can circumvent the deleterious effects of body fluids (e.g. blood serum). The procedure encompasses the following steps: (i) capture of analyte molecules from serum by MP-antibody conjugates, (ii) pelleting of MP-antibody-analyte complexes, using a magnetic field, followed by exchange of serum for optimized biological buffer, (iii) mixing of MP-antibody-analyte complexes with actin filaments conjugated with same polyclonal antibodies as the magnetic nanoparticles. This causes complex formation: MP-antibody-analyte-antibody-actin, and magnetic separation is used to enrich the complexes. Finally (iv) the complexes are introduced into a nanodevice for specific binding via actin filaments to surface adsorbed molecular motors (heavy meromyosin). The number of actin filaments bound to the motors in the latter step was significantly increased above the control value if protein analyte (50–60 nM) was present in serum (in step i) suggesting appreciable formation and enrichment of the MP-antibody-analyte-antibody-actin complexes. Furthermore, addition of ATP demonstrated maintained heavy meromyosin driven propulsion of actin filaments showing that the serum induced inhibition was alleviated. Detailed analysis of the procedure i-iv, using fluorescence microscopy and spectroscopy identified main targets for future optimization.

**Conclusion:**

The results demonstrate a promising approach for capturing analytes from serum for subsequent motor driven separation/detection. Indeed, the observed increase in actin filament number, in itself, signals the presence of analyte at clinically relevant nM concentration without the need for further motor driven concentration. Our analysis suggests that exchange of polyclonal for monoclonal antibodies would be a critical improvement, opening for a first clinically useful molecular motor driven lab-on-a-chip device.

## Background

In the recent decades, healthcare costs have soared throughout the industrialized world and this development is predicted to continue, e.g. with the costs reaching 30% of the gross domestic product in the US 2035 (compared to 15% in 2007 and 5% in 1960 [1]) Together with environmental and climate issues this is one of the biggest challenges facing industrialized nations. The efforts to develop new biosensing devices that are cheaper, faster, and more accurate should be viewed in this context. Such devices would allow detection of diseases and environmental changes at an early stage with increasing chances for interventions at a low cost.

Of interest in this connection are lab-on-a-chip devices [2-4] where miniaturized chips, can perform a series of analyses and be used at the point of care or in the field rather than in a centralized laboratory. However, while appreciable progress has been made towards such devices [3] they often require expensive and bulky accessory equipment [3,4]. For instance, pumps that drive microfluidics flow demand increasingly more power the greater the miniaturization [5] and the manufacturing of the chip components becomes increasingly challenging and expensive. To overcome these problems it has been proposed that biological molecular motors, with their inherent extensive miniaturization, biodegradability and self-propelling features, may be used to transport analytes e.g. from recognition to detection chambers, achieving separation, concentration as well as certain forms of detection [6-8]. Several important steps towards a functional molecular motor driven diagnostic device have also been realized (reviewed in [8-13]) such as: (i) attachment of antibodies to cytoskeletal microtubule [14] and actin filament [15] shuttles, followed by molecular motor-driven transportation of analytes (viruses, protein antigens etc.) bound to the antibodies, (ii) nano/microfabrication of devices for guided transportation of the motor propelled shuttles to concentrate analytes at a detector site [6,7,16-18] and (iii) long-term storage of ready-to-use devices without loss of activity [19-21].

Despite the above developments, challenges remain before a commercially viable molecular motor driven device is realized. Particularly, we showed recently [22] that complex fluid environments, such as blood plasma, blood serum and cell lysates may have deleterious effects on molecular motor driven propulsion of both actin filaments and microtubules, unless the samples are diluted > 100 times. These problems are essential to overcome since extensive sample dilution is highly undesirable in high-sensitivity detection. Separation of targeted molecules from biological fluids, using magnetic microparticles with antibodies immobilized on their surface, may be useful in this connection. Such separation is a technique of growing importance in biosensing [23,24] and more recently, also magnetic nanoparticles have been used for similar purposes [25].

Here we utilize magnetic nanoparticles to investigate whether an approach with a magnetic pre-separation step is a way forward in alleviating the deleterious effects of complex fluid environments on motor driven diagnostics devices. Our results are promising, showing effective exchange of the deleterious fluid components for optimized biological buffers thus enabling subsequent motor driven transportation. We also found evidence that the presence of antigen (analyte) cross-links magnetic particles and actin filaments to an appreciable degree if both particles and filaments are conjugated with polyclonal antibodies against the analyte in question. Whereas the method is potentially useful already at the present stage of development, our microscopy and spectroscopy based analysis suggests that there is appreciable room for further optimization as discussed in some detail.

## Results and discussion

**Figure 1:**
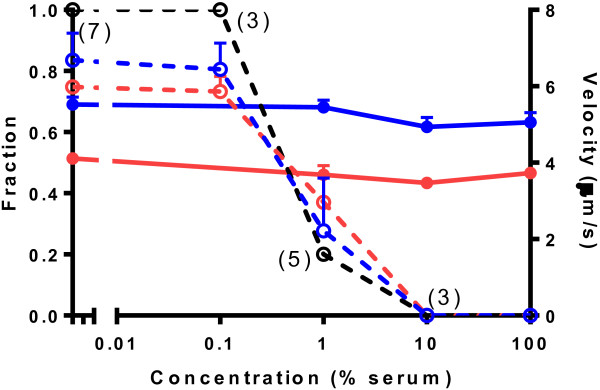
**Effects of serum on actomyosin motility with and without magnetic separation to remove serum components. **Full symbols and lines: with magnetic separation. One control experiment but at least twenty filaments for each velocity measurement. Dashed symbols and lines: without magnetic separation. Number of independent experiments in parentheses. Fraction of motile filaments (red; left vertical axis), average sliding velocity (blue; right vertical axis) and the fraction of experimental occasions with any motility at all (black) when the flow cells were directly incubated with blood serum at different dilutions. Sliding velocity (blue dashed line and open circles; data adapted from [22]). Error bars show SEM.

Molecular motor-based proof-of-principle nanodevices for diagnostics applications [6,7,16,26] have been tested in optimized biological buffers but not in complex fluid environments, such as blood serum, encountered in a real device. This is a concern as we recently [22] found that both actomyosin and microtubule-kinesin sliding velocity is effectively reduced or inhibited by body fluids (blood components, cell lysates) unless diluted 100–200 times. This finding is expanded on in Figure 1 where we, in addition to effects on velocity, also show concentration-dependent effects of serum on the fraction of motile filaments and on the fraction of independent experiments where actomyosin motility was observed. Different factors are likely to contribute to the deleterious effects. In addition to serum proteins such as gelsolin, directly depolymerising the actin filaments [27], there may be binding of other blood serum components to HMM or actin [22].

**Figure 2:**
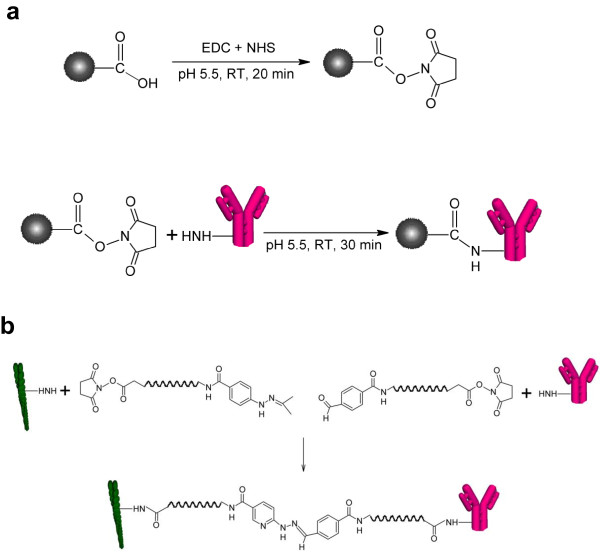
**Schematic representations of the conjugation reactions. **(**a**) Preparation of anti rabbit IgG coupled magnetic nano particle. Carboxy (COOH) functionalized magnetic nanoparticles are activated with EDC and NHS. Amine-reactive NHS ester on the magnetic nanoparticle reacts with primary amines on the antibody to yield anti rabbit IgG coupled magnetic nano particle. (**b**) Schematic representation of the conjugation reaction between F-actin and anti rabbit IgG using heterobifunctional cross-linkers. F-actin is modified with C6-succinimidyl 6-hydrazinonicotinate acetone hydrazone (C6-SANH) via primary amines on actin which then forms a biz-aryl-hydrazone bond with C6-succinimidyl 4-formylbenzoate (C6-SFB) attached to anti rabit IgG via primary amines.

**Figure 3:**
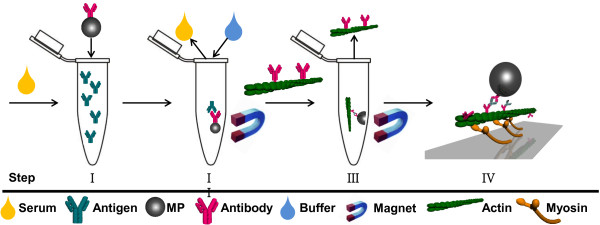
**Principle for magnetic pre-concentration procedure. **Whereas the antibody in the present study was anti-rabbit IgG and the antigen, rabbit IgG, the terms “antibody” and “antigen” are used to illustrate the generality of the approach. In step I, antigens are captured from serum by the antibodies conjugated to magnetic particles, MPs. Antigen-antibody-MP complexes are then concentrated using an external magnet to a small volume in the eppendorf tube while simultaneously exchanging serum for an optimized biological buffer (step II). Actin filaments conjugated with antibodies are then added and a second magnetic concentration step is performed (step III). This step is expected to leave actin filaments without MPs in the supernatant (that is removed) and those that have cross-linked MPs in the pellet. The latter are re-dispersed in buffer B and added to a flow cell (step IV) with surfaces coated with heavy meromyosin motor fragments for specific binding of actin filaments (e.g. actin-antibody-antigen-antibody-MP complexes as illustrated) and molecular motor driven transportation.

To overcome the deleterious effects of blood serum we here report a process based on magnetic separation that allows exchange of body fluids for optimized biological buffers without a dilution step. This process is unique in that it encompasses separation steps relying on both antibody-antigen interactions, magnetic forces and the specificity of the actomyosin bond. In this method we used commercially available ferromagnetic metal nanoparticles (MP) coated with a thin layer of carboxy (COOH) functionalized carbon graphite. Following 1-(3-Dimethylaminopropyl)-3-ethylcarbodimide hydrochloride (EDC) and N-Hydroxysuccinimide (NHS) incubation to create amine-reactive NHS ester groups on the MPs, the particles were linked to primary amines on the antibodies (Figure 2(a)). Actin filaments with covalently attached antibodies were obtained [15] using hetero-bifunctional cross-linkers (Figure 2(b)). The separation workflow (see also Methods) is illustrated in Figure 3. First, MPs (< 50 nm diameter) conjugated with polyclonal anti-rabbit IgG (a-rIgG) antibodies were used to harvest the model analyte (rabbit-IgG;rIgG) from serum (Figure 3:I) followed by magnetic separation and exchange for a standard motility buffer (Figure 3:II). The MPs with antibody-antigen complexes were then (Figure 3:III) mixed with Alexa-488 phalloidin (APh) labeled actin filaments, conjugated with polyclonal a-rIgG [15]. Next, a 30 min incubation period allowed formation of antibody-analyte-actin complexes e.g. actin-(a-rIgG)-(rIgG)-(a-rIgG)-MP aggregates. This was possible in our experiments due to the polyclonality of the a-rIgG antibody. Following an additional magnetic separation step the aggregates were enriched and transferred to a flow cell (simulating a diagnostic nanodevice) where they were tethered to myosin motor fragments (heavy meromyosin; HMM; Figure 3:IV). This final step relies on the specific recognition of the actin filaments by the myosin motor domains, thus adding specificity to the process.

**Figure 4:**
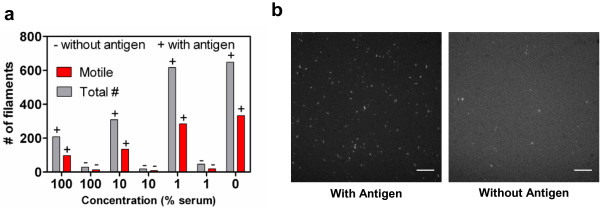
**Effects of magnetic pre-concentration and removal of serum. **(**a**) The total number of filaments (grey bars) and the number of motile filaments (red bars) at the end of the procedure in Figure 3 for different concentrations of blood serum (0 – 100%) and in the presence (+) and absence (-) of antigen (r-IgG) in step I in Figure 3. (**b**) Fluorescent images from the experiment in (**a**) with or without antigen. In the presence of antigen (rIgG) several fluorescent filaments can be seen suggesting crosslinking between arIgG antibodies conjugate on actin and MPs via the antigen. In the absence of antigen only a few filaments are bound to HMM on the surface. Both images are from samples with 100% serum. Scale bar 10 μm.

The successful serum elimination is indicated by our findings that the fraction of motile filaments and the velocities of HMM propelled actin filaments in the presence of 1 mM MgATP, were independent of the serum concentration during the initial incubation step (Figure 1, full lines; see further below). The number of filaments, that were transferred to the flow cell (Figure 4(a)) and that bound to HMM in the final step in Figure 3, was significantly higher (p = 0.048) in the presence of rIgG (60 nM) in the serum incubation step (378 ± 123 per image frame, n = 3 flow cells) than in its absence (31 ± 8 per image frame, n = 3). Moreover, a similar fraction of motile filaments (40–50%; Figure 4(a), red bars) and similar velocities (not shown) were seen in the absence and presence of rIgG pre-incubation. We attribute these data (Figure 4(a) and 4(b)) to: (i) enrichment of actin filaments in the second magnetic separation process (step III in Figure 3) indicating cross-linking of actin filaments to MPs via rIgG, (ii) effective binding of the aggregates to the HMM coated surface and (iii) lack of motility inhibition by MPs (as motility quality was not altered after the magnetic separation steps). The lower total number of observed filaments in 10 and 100% serum in Figure 4(a), compared to more extensive serum dilutions, is consistent with inhibiting effects of serum on the binding between a-rIgG and rIgG. Such effects are expected for a multiplicity of reasons [28]*e.g*. non-specific binding of other proteins to the antibodies.

The above results were confirmed in two additional independent experiments (using different batches of conjugated actin and conjugated MPs with rIgG as antigen). In these experiments an incubation step with bovine serum albumin was introduced just before addition of actin filaments (see Methods). However, this did not cause any apparent changes in the results compared to those in Figure 4. Therefore data from all experiments in buffer and 100% serum (with and without antigen) were pooled from the two sets of experiments. The results from these pooled experiments are summarized in Table 1. In accordance with the results in Figure 4, there was no significant difference in velocity and fraction of motile filaments between the optimized biological buffer, the serum sample without antigen and the serum sample with antigen. However, the presence of antigen during the incubation led to a statistically significant increase in the number of HMM-bound filaments, in step IV in Figure 3. Similar results were obtained whether the filament number was obtained by direct counting or if the relative filament density under different conditions was estimated from total background-subtracted fluorescence intensity (Table 1). Whereas the latter method exhibited increased variability it was considerably faster. The results in Table 1 are also fully consistent with results using Rhodamine-labelled rIgG (Rh-rIgG) as antigen (Additional file 1: Figure S1).

**Table 1:** **Effect of antigen (Ag) on number of actin filaments, fluorescence intensity and actomyosin motility quality in final stage of the magnetic separation procedure in Figure **3

**Sample**	**Velocity (μm s**^**-1**^**)**	**Fraction Motile**	**# of filaments**	**Intensity (AU)**
Buffer B with Ag (n = 3)	6.44 ± 0.48	0.51 ± 0.03	628 ± 38	0.89 ± 0.11
Serum (100%) with Ag (n = 3)	5.86 ± 0.44	0.38 ± 0.05	523 ± 164	0.81 ± 0.11
Serum(100%) without Ag (n = 3)	6.26 ± 0.71	0.27 ± 0.08	27 ± 11*^1^	0.22 ± 0.07*^2^

*p < 0.05; ^1 ^p = 0.0256, ^2 ^p = 0.0281 (normalized). AU: normalized arbitrary units.

Sample composed of buffer B or 100% serum with (50-60 nM) or without rIgG (Ag) was added at the first stage of this procedure. Data given as mean ± standard error of the mean from three independent experiments for each condition. Intensity values are given as normalized arbitrary units since different fluorophores were used in the different experiments. Repeated measures one-way Analysis of Variance (ANOVA) was used for statistical hypothesis testing.

The rather low fraction of motile filaments in the experiments in Figure 1, 4 and Table 1 is due to (i) omission of the incubation step with blocking actin (non-fluorescent actin at 1 μM concentration) often used to block ATP-insensitive myosin heads, and (ii) omission of a step with actin affinity separation by ultracentrifugation (to remove the ATP-insensitive heads) prior to the motility assay. The first of these omissions was important in order to leave as many myosin heads as possible free to bind actin filaments.

There was a tendency for a particularly low fraction of motile filaments in step IV (Figure 3) in absence of antigen in the sample solution (lower row, Table 1). The basis for this finding can be sought in the very low number of short actin filaments (Figure 4b) under these conditions. Whereas, the low number is due to lack of cross-linking of actin filaments to MPs in the absence of antigen, we attribute the fragmentation of the filaments to shearing forces e.g. related to repeated mixing and pipetting. The short filaments detach from the surface with higher probability than long filaments but only if they are motile and not if they are bound to rigor-like ATP-insensitive heads. This leads to an appreciable effect on the fraction of motile filaments particularly when the total number of filaments is low as was the case in the initial absence of antigen.

For increased specific cross-linking efficiency between MPs and actin filaments we attempted to use magnetic microparticles (average size 1 μm, according to manufacturer) instead of nanoparticles. However, due to the high binding capacity of the larger particles there was an appreciable tendency for formation of large aggregates composed of both filaments and microparticles (Additional file 1: Figure S2). These large aggregates may be potentially useful for magnetic concentration of actin-MP aggregates but are clearly not useful in separation schemes where actomyosin driven transport is required, e.g. for motor driven concentration [7,29], subsequent to the magnetic pre-separation. In this case, nanoparticles must be used. Their considerably smaller surface area (although not smaller surface/volume ratio) make them less sticky consistent with the findings that actomyosin driven motility is less affected by large than small nanoscale cargoes [30]. Furthermore, the small size of nanoparticles prevents clogging of micro and nanoscale channels [31,32] of the type that we have generally used to guide HMM propelled actin filaments. The increase in the number of actin filaments on the surface (Figure 4, Table 1) at the end of the procedure in Figure 3, in itself, reports the presence of analyte whether the filaments are cross-linked to MPs or not. Just by counting the number of actin filaments or measure the increase in fluorescence intensity due to actin filament binding to HMM on the surface we could detect the presence of analyte in the 10–100 nM concentration range (Additional file 1: Figure S1). This is in the relevant range for several clinically important disease markers, e.g. the inflammation marker C-reactive protein (CRP) [33]. However, while detection of 50–60 nM antigens gave consistent results between experiments in terms of the number of observed filaments in step IV of Figure 3, attempts to consistently detect antigen at lower concentration (0.5 - 5 nM) was unsuccessful (Additional file 1: Figure S1) as further discussed below.

To summarize, we have demonstrated a conceptually useful approach for capturing analytes from serum for subsequent motor driven separation/detection. The usefulness of the method was clear from the consistent signal in the final step in Figure 3 (corresponding to a certain number of actin filaments) for a sample antigen concentration of 50–60 nM. However, clearly, our results also suggest that there is appreciable room for improvement. In order to understand complicating factors to facilitate future optimizations, we used a combined microscopic and fluorescence spectroscopic approach to analyze the different steps in the procedure in Figure 3.

**Figure 5:**
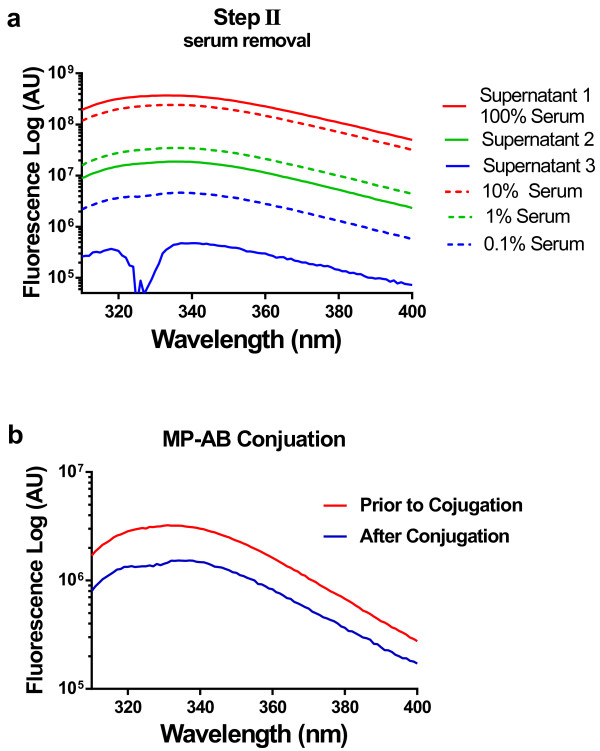
**Fluorescence spectroscopic monitoring of the separation process. **(**a**) Tryptophan fluorescence from the supernatants during different magnetic washes in step II (Figure 3) where serum is removed. MPs and bound antigens are pelleted with an external magnet while serum is removed (red full line). The pellet is then resuspended in 1 ml buffer A and then pelleted again while the supernatant was removed (green full line). This washing procedure was repeated two more times (blue full line). Dashed lines show (for reference) dilutions of serum to 10% (red), 1% (green) and 0.1% (blue). (**b**) Tryptophan fluorescence from the solution before and after the conjugation between MPs and antibodies. Red line: emission from antibody sample before magnetic particles were added. Blue line: emission from the supernatant after conjugation reaction where magnetic particles and bound antibodies are pelleted using a magnet.

First, of critical importance is insight into the effectiveness of the selective removal of serum components in step II in Figure 3. This step was monitored by measuring the tryptophan fluorescence (Figure 5a) from the supernatants after magnetic pelleting. As exemplified in Figure 5a (similar in one further experiment) there was < 1% serum proteins left in the supernatant after a first washing sub-step (within step II) as indicated by the tryptophan fluorescence. Here, we studied the supernatant rather than the MP-containing pellet due to disturbing effects of the MPs on the fluorescence signal. Altogether, two further washes, each associated with magnetic pelleting and exchange of supernatant, in step II, ensured that negligible amounts of serum proteins (<0.1% ) remained when the MP-Ab-Ag conjugates were mixed with the antibody-conjugated actin filaments in the next step (step III in Figure 3). This finding is consistent with the observation (Figure 1) of maintained actomyosin function after the entire procedure in Figure 3. However, the spectroscopic data show that the removal of serum in step II is actually appreciably more effective than can be inferred from the lack of effects on motility. Indeed, already the degree of serum removal seen after one magnetically assisted washing step would be sufficient [22] to account for the finding that motility was unaffected (Figure 1). The very effective serum removal has important implications. First, it makes it very unlikely that actin binding proteins in serum [27], *e.g*. the severing protein gelsolin contribute to the observation of small, apparently fragmented filaments in the final step in Figure 3. Instead, we attribute the latter observation to shear forces that affect the filaments during repeated pipetting, mixing and, possibly, the magnetic separation itself (Figure 3, step III; Additional file 1: Figure S3). Second, the finding of very effective serum removal suggests that 1–2 washes would be sufficient in steps II and III rather than 3 and 5 washes, respectively, used here. This would have the additional effect to reduce shearing forces on the actin filaments.

For further optimization, it is of interest to know to what extent that antigens are actually captured by the antibody-conjugated MPs in step I of the procedure in Figure 3. First, in order to quantify the number of antibodies per MP in the reaction in Figure 2a we measured (Figure 5b) the intrinsic protein fluorescence prior to, and after coupling. The decrease in the antibody concentration in the MP-free solution suggested by these measurements indicated antibody coupling corresponding to about 40 antibodies per MP i.e. an antibody concentration of 1.2 μM assuming a MP concentration of about 30 nM in step I of the procedure in Figure 3.

**Figure 6:**
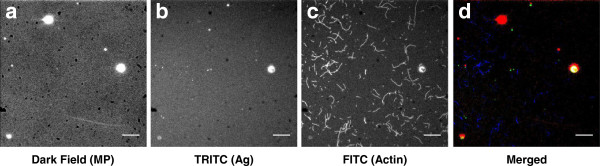
**Aggregation of magnetic microparticles observed on a flow-cell surface pre-incubated with HMM and a-rIgG conjugated actin filaments (after step I in Figure **3**). **(**a**) Dark field microscopy where bright spots are believed to represent MPs or aggregates of several MPs. (**b**) Fluorescence micrograph using TRITC filter set to visualize Rh-rIgG (**c**) Fluorescence micrograph using FITC filter set to visualize APh labeled actin filaments. (**d**) Merge of (**a**)-(**c**) with dark-field images red, Rh-rIgG (TRITC) green and actin filaments (APh) blue. Scale bar 10 μm.

Most likely, (cf. [34]) the concentration of antibodies with antigen binding capacity (i.e. those that are appropriately oriented and in native state) was considerably lower. Additionally the degree of antigen binding is likely to be reduced if aggregates of MPs are quickly formed via antibody-antigen links. The formation of such links is possible with the polyclonal antibodies that we used here. This effect would be particularly severe at low concentrations of antigen where only a small fraction of the antibodies on the MPs are occupied, leaving a large number of them available for cross-linking to other antibody conjugated MPs. This effect, illustrated in Additional file 1: Figure S4, would also cause appreciably reduced cross-linking between MPs and antibody conjugated actin filaments when the antigen concentration is low relative to the concentration of antibody-conjugated MPs. The reason is that majority of the antigens, under these conditions, would be hidden inside MP-MP aggregates as indicated in Additional file 1: Figure S4. This is in good agreement with the lack of consistent detection of antigen at concentrations of 5 nM or less. That MP-antigen aggregates did really form was demonstrated by direct microscopic observation (Figure 6). Thus, after performing step I (50 nM antigen, 10 min) in Figure 3 we took out a sample that was infused into a flow cell placed on a magnet (the one used for magnetic separation) during a 30 s incubation period. Now, using dark-field and fluorescence microscopy we observed several, micrometer sized aggregates that we attribute to magnetic particles with captured Rh-rIgG molecules.

We also searched for similar aggregates in step IV (Figure 3), i.e. after completion of the entire procedure but very few were observed (data not shown) in spite of a large number of actin filaments compared to the control situation. Provided that all MPs could be detected by the combined dark-field and fluorescence microscopy, this suggests that very few MPs are required for an appreciably increased number of actin filaments in step IV.

Therefore, the consistent increase in the number of actin filaments at 50 nM antigen, is strong support of the usefulness of the described approach. Furthermore, it would be trivial to substitute polyclonal antibodies with monoclonal antibodies in a real device thereby eliminating complexities due to MP-MP aggregate formation. In the case with monoclonal antibodies, those on the magnetic particles would be directed against another epitope of the antigen than those on the actin filaments. In addition to increased sensitivity, this approach would also increase the specificity due to the involvement of two different specific antibodies in creating the diagnostically relevant link between magnetic particle and actin filament.

Sensitivity and detection rate should be possible to increase further by substituting filament counting and whole frame intensity measurements by an automated process whereby a concentrator device [7,29] concentrates the motile actin filaments in a nanoscale detector area. This would give a measure of the fluorescence intensity attributed to HMM propelled actin filaments insensitive to e.g. changes in background fluorescence or altered illumination conditions that may severely affect the full-frame fluorescence intensity [35]. Moreover, the concentration approach is automated and fast compared to the manual filament counting procedure.

A third modification of the procedure that is likely to cause significant improvements, both with regard to limit of detection and specificity, would be the use of magnetic forces to pull actin-MP aggregates to the surface in the final step in Figure 3. In this case, only those actin filaments that are linked to MPs would be pulled by the magnetic field whereas only those MPs that are bound to an actin filament would be specifically linked to the surface via HMM. Under these conditions the flow cell height should be increased, from the current value of 0.1 mm, in order to increase the volume from which actin-MP aggregates are recruited and increase the ratio of magnetically driven to diffusional transport.

As indicated above, the procedure in Figure 3 would be readily interfaced with a molecular motor driven concentrator device [29,36] for additional 100–1000 fold signal amplification. Finally, we have presented evidence that the number of rinsing steps may be appreciably reduced. Also the incubation times may be reduced. For instance only 10 min incubation period was used in a recent immunoassay [24] for binding of analyte at attomolar concentration to magnetic microparticles in solution. However, optimizations of the detailed assay procedure would require a large number of time consuming tests. These would not add much of conceptual interest and are therefore outside the scope of the present study.

## Conclusions

We have described a novel approach for capturing analytes from serum by antibody conjugated magnetic particles adapted for use in molecular motor driven concentrator devices [7,29]. Importantly, this method eliminates the recently discovered [22] deleterious effects of serum on the actin filaments and on actomyosin motor function. Subsequent to the analyte capture, magnetic separation thus allows exchange of body fluids (e.g. blood serum) for an optimized biological buffer and subsequent mixing with antibody-conjugated actin filaments. Now, large aggregates tend to form between actin filaments and micrometer sized antibody coated particles, opening for conventional magnetic concentration of aggregates between actin filaments and magnetic particles. On the other hand, magnetic nanoparticles form smaller aggregates with actin filaments that may be transported by heavy meromyosin motor fragments. Pending optimizations, particularly the use of monoclonal antibodies as suggested by our analysis of the described procedure, opens for use of a molecular motor driven concentrator device to achieve further signal amplification.

## Methods

### Materials

Magnetic nanoparticles (MPs) with mean diameter of 30 nm (according to manufacturer) and surface-coated with primary carboxylic groups was purchased from TurboBeads (Zurich, Switzerland). C6-succinimidyl 6-hydrazinonicotinate acetone hydrazone (C6-SANH) and C6-succinimidyl 4-formylbenzoate (C6-SFB) were purchased from Solulink, San Diego, CA, USA. Anti-rabbit IgG (H&L, Goat, a-rIgG) was purchased from Rockland Immunochemicals, Gilbertsville, USA. Zeba desalt spin columns and bicinchoninic acid (BCA) protein assay kits were purchased from Pierce Rockford, IL, USA. Rabbit IgG (rIgG) and all other chemicals were of analytical grade and, unless otherwise stated, purchased from Sigma-Aldrich Sweden AB, Stockholm, Sweden.

### Preparation of muscle proteins

Actin was prepared from rabbit skeletal muscle [37] and actin filaments were fluorescently labeled with Alexa-488 phalloidin (APh) (Molecular Probes Invitrogen, Eugene, OR) [32,38,39]. Myosin II was purified from rabbit fast skeletal muscle and heavy meromyosin (HMM) was prepared by digestion of myosin with α-chymotrypsin [38,40]. All experiments using animal material were performed in accordance with national and EU-legislation and were approved by the Regional Ethical Committee for Animal experiments (reference # 96–11), Linköping, Sweden.

### Activation and coupling of magnetic nanoparticles

Magnetic nanoparticles (30 mg mL^-1^ corresponding to 83 nM; according to manufacturer´s specifications) were dispersed by sonication in an ultrasonic bath for 15 minutes, then collected using a magnet (Neodymium Magnet, 14 × 8 mm, pulling strength 5 ± 0,5 kg. Svenska Magnet Fabriken AB, Hallstahammar, Sweden) and washed with 1 mL of activation buffer (54 mM (2-(*N*-morpholino)ethanesulfonic acid (MES), pH 5.5). These steps were repeated three times. One mL of MP solution (30 mg mL^-1^ in activation buffer) was mixed with 1 mL of activation solution (10 mg EDC and 10 mg NHS dissolved in 1 mL activation buffer) and incubated for 20 min at room temperature (RT) on a shaker. The activated MPs were collected using the magnet and the supernatant was discarded. One mg of a-rIgG was mixed with 1 mL of the activated MPs in activation buffer and incubated for 30 minutes at RT on a shaker. This was followed by washing four times with 1 mL of PBST (10 mM sodium phosphate, 0.15 M NaCl with 0.5% Tween-20, pH 7.4) and finally two times with 1 mL of buffer A (1 mM MgCl_2_, 10 mM 3-morpholinopropane-1-sulfonic acid (MOPS), 0.1 mM K_2_-ethylene glycol tetraacetic acid (EGTA), pH 7.4). The a-rIgG-conjugated MPs were then mixed with 1 mL of storage buffer (buffer A with 0.05% sodium azide) and stored at 4°C over night.

### Preparation of anti-rabbit IgG conjugated actin filaments

F-actin, conjugated with a-rIgG was prepared essentially as described previously [15]. First, F-actin (3.5 mg mL^-1^ in modification buffer 1; 100 mM (4-(2-hydroxyethyl)-1-piperazineethanesulfonic acid) (HEPES), 150 mM KCl, 5 mM MgCl_2_, 1 mM Na_2_ATP, pH 8.0) was incubated with C6-SANH linker (dissolved in Dimethyl sulfoxide, DMSO) at RT for 2 h with a molar ratio of 1:2 (actin:linker). Excess linker was removed using ultracentrifugation (Beckman-Optimamax-XP ultracentrifuge, 100,000 g, 4°C, 25 min) through a 10% glycerol cushion. Pellets were re-suspended in modification buffer 1 with 5% glycerol cushion. A second ultracentrifugation step was applied with subsequent re-suspension in conjugation buffer (100 mM MES, 150 mM KCl, 5 mM MgCl_2_, 1 mM Na_2_ATP, pH 6.0). The actin solutions were sonicated gently on ice to disperse the filaments and these were then stored on ice until the conjugation with a-rIgG. Our previous results [15] suggest that the covalent modification of actin with the C6-SANH linker does not modify actin function.

The a-rIgG (2 mg mL^-1^ in PBS buffer; 0.1 M sodium phosphate, 0.15 M NaCl, pH 7.4) solution was incubated with 10-fold molar excess of C6-SFB linker (dissolved in DMSO) at RT for 2 h. Modified a-rIgG was desalted using zeba desalt spin column against conjugation buffer (see above) to remove excess linker and to exchange buffer. The modified antibodies were stored on ice until further use.

The C6-SANH modified F-actin sample was incubated with C6-SFB modified a-rIgG solutions. Addition of 10 mM catalyst buffer from stock solution (100 mM aniline in conjugation buffer) started the reaction that was allowed to proceed for 6 h at RT. The molar ratio of modified F-actin and modified a-rIgG was 2:1 respectively.

In order to remove catalyst and exchange the buffer, samples were dialyzed at 4°C against G-actin buffer (2 mM Tris base, pH 8.5, 0.2 mM Na_2_ATP, 0.5 mM dithiothreitol (DTT), 0.2 mM CaCl_2_, 3 mM NaN_3_). For long-term storage, samples were flash frozen in liquid nitrogen and stored at -80°C.

The conjugated G-actin monomers (actin-a-rIgG) were co-polymerized with non-conjugated monomers to form filaments with 1:3 molar ratio of conjugated to non-conjugated actin. The co-polymerization reaction was performed at 4°C (3 h) by addition of KCl, MgCl_2_ and ATP to final concentrations of 100 mM, 2 mM and 3.3 mM, respectively. The co-polymer was fluorescence-labeled with APh or RhPh (molar ratio: 1:1.5; actin:APh or RhPh) at 4°C over night (see above).

### Magnetic capture of analytes from serum

Figure 3 illustrates the principle for magnetic capture of antigen from blood and the subsequent separation from other serum components and integration with the actomyosin motor system. Serum was first obtained from human blood drawn into Vacutainer™ glass tubes. The blood was from volunteers who consented to the procedures under a protocol approved by the regional ethical committee in Linköping, Sweden. After 1 h at RT the sample was centrifuged (3000 g for 20 minutes at RT; 20°C) to remove blood cells and solidified fibrin thus giving blood serum. Three different concentrations of serum were prepared by dilution in buffer B (buffer A with 1 mM DTT and 50 mM KCl): 100%, 10% and 1%. For the subsequent magnetic separation process the different serum samples were aliquoted into six (two for each concentration), tubes (200 μl per tube). Magnetic particles conjugated with a-rIgG were added to all six tubes (2.5 mg magnetic particles per tube).

One additional tube contained 0% serum (100% buffer B). The rIgG (60 nM) was added to four of the vials (100%, 10%, 1% and 0%) and all seven tubes were put on a shaker for 1 h at RT. Samples were then washed 10 times each with 500 μl of buffer B while applying magnetic forces to keep magnetic particles and their bound analytes (rIgG) in the tubes. This allowed removal of serum and exchange for optimized biological buffer (buffer B). Each tube was then incubated with 250 nM a-rIgG conjugated actin filaments (final volume 250 μl) for 1 h at RT on a shaker in the dark followed by gentle wash 10 times in buffer B under magnetic separation forces. Finally 250 μl of buffer B was added to the tube before the content was infused into a motility assay flow cell.

In another set of experiments, 2.5 mg MP-a-rIgG was mixed with 100% Serum and 0% Serum with 50 nM of rIgG for 30 min at RT on a shaker in the test samples. Alternatively, 0.5-50 nM Rh-rIgG was used as antigen. Control samples were treated similarly but were without rabbit IgG. After stipulated times the samples were washed three times with 1 mL of PBST (not used with Rh-rIgG) and three times with 1 mL of buffer A, while applying magnetic forces to keep magnetic particles and bound analytes (rIgG) in the tubes. This was expected to allow effective removal of serum components. Subsequently, 300 μL of 1% bovine serum albumin (BSA) was added to each tube followed by 30 min incubation at RT under gentle shaking. This step when used (not with Rh-rIgG) was followed by two times wash with 1 mL of buffer A. Each tube was then incubated with 250 nM a-rIgG conjugated filaments for 30 min at RT on a shaker in the dark. Samples were then washed gently seven times (five times with Rh-rIgG) in buffer B under magnetic pelleting of complexes with magnetic particles, e.g. MP-a-rIgG-rIgG-a-rIgG-actin and final addition of 250 μl buffer B to re-disperse the pellet (see above).

### In vitro motility assays (IVMA)

Flow cells were constructed from two cover-slips with double-sided sticky tape as spacers. The motility supporting surface (bottom of flow cell) was derivatized with trimethylchlorosilane (TMCS) as described previously [41]. All solutions that were added to the flow cell were based on buffer A (see above) and all proteins were diluted in buffer B (see above). The flow cell was pre-incubated according to standard procedures [15,39,42]: (i) HMM (120 μg mL^-1^) for 2 min, (ii) 1 mg mL^-1^ BSA for 30 s (iii) wash with buffer B and (iv) addition of re-dispersed pellet from the magnetic separation (described above). After an incubation period of 3 min, flow cells were washed with buffer B, and (vi) incubated with r60 assay solution (buffer A with 10 mM DTT, 35 mM KCl, ionic strength 60 mM). The r60 solution was also supplied with an anti-bleach system (final activity concentrations of 3 mg mL^-1^ glucose, 20 U mL^-1^ glucose oxidase and 870 U mL^-1^ catalase). Finally, (vii) a60 assay solution was added to the flow cell to induce filament sliding. The a60 solution was similar to the r60 solution but with addition of 1 mM MgATP and an ATP regenerating system (2.5 mM creatine phosphate and 3.5 U mL^-1^ creatine phosphokinase).

### Fluorescence spectroscopy

Fluorescence spectroscopy was performed using a FluoroLog FL3-22 Spectrophotometer (Instruments S.A Inc. New Jersey, USA). For estimation of changes in antibody and serum protein concentrations, we used an excitation wavelength of 295 nm (slit 2 nm) and read emission in the range from 310 nm to 400 nm (slit 6 nm). Antigens labeled with rhodamin (~2 rhodamin per antibody) were studied using an excitation wavelength of 555 nm (slit 3) and emission was read at 570–700 nm (slit 5 nm).

### Microscopy

Fluorescently labeled actin filaments were observed using a Nikon Eclipse TE300 inverted fluorescence microscope (Nikon Corp., Tokyo, Japan) equipped with a temperature-regulated Nikon (100 × 1.4 NA) oil immersion objective, TRITC (Ex. 540/25, DM 565, and BA 605/25) and FITC (Ex. 465–495, DM 505, and BA 515–555) filter sets. Image sequences were recorded using a cooled Hammamtsu EMCCD camera as described previously [32]. Actin velocities were measured using a manual tracking program developed in a Matlab enviroment (The MathWorks Inc, Natick, MA) [43,44]. The number of actin filaments and the total intensities of the different frames were measured using Image J (Rasband, W.S., ImageJ, U. S. National Institutes of Health, Bethesda, Maryland, USA, http://imagej.nih.gov/ij/, 1997–2012.).

Dark field microscopy was performed using a Nikon oil immersion dark field condenser together with a Nikon oil immersion objective (100 ×, 0.5-1.3 NA). The latter was also used for fluorescence microscopy in Figure 6.

### Statistical and related analysis

Statistical analyses were implemented in GraphPad Prism (v. 6.01; GraphPad Software, San Diego, CA). Unless otherwise stated, data are given as mean ± standard error of the mean (SEM) and statistical hypothesis testing was performed using two-tailed t-test (paired when appropriate) or repeated measures ANOVA.

Images from the individual experiments were analyzed manually by counting the number of filaments in one frame from each flow cell. These filaments were then categorized into stationary or motile by observation in 25 frames (5 seconds of real time). To verify the manual counting method the average background-subtracted intensity from 50 consecutive image frames of stationary filaments was also obtained and used as an alternative measure of the relative number of filaments. Average background pixel intensity was calculated as the mean pixel intensity value in four filament-free areas, one in each quadrant, of the averaged intensity image.

## Competing interests

AM is a co-founder, co-owner and CEO of the start-up company ActoSense Biotech AB (Kalmar, Sweden) aiming to develop diagnostic devices based on the aggregation of cytoskeletal elements, particularly actin filaments, in solution. Moreover, AM holds two Swedish patents in this field and application for one of these patents (about aggregation of actin filaments by analyte molecules) has also been filed in the US and Europe. Finally, AM, SK and LtS have applied for a Swedish patent related to this work.

## Authors’ contributions

AM and SK laid out the foundations of the project. SK, LtS, MP and NAT performed the experiments. LtS, SK, AM, NAT and MP analyzed the experiments. AM, LtS, SK wrote the article. All authors read and approved the final manuscript.

## Supplementary Material

Additional file 1: Figures S1-S4 Figure S1 shows titrations where the number of HMM bound actin filaments (step IV, Figure 3) is shown as a function of antigen concentration (0.5-50 nM). Figure. S2 shows a fluorescent micrograph illustrating aggregation of magnetic microparticles. Figure S3 and Figure S4 show schematic illustrations of filament fragmentation (S3) and magnetic nanoparticle aggregation (S4). Click here for file
