# Supplementary material for: Magnetic capture from blood rescues molecular motor function in diagnostic nanodevices
Source: J Nanobiotechnology. 2013 May 3;11:14. doi: 10.1186/1477-3155-11-14 (PMC3660291; doi:10.1186/1477-3155-11-14)
Supplement: Additional file 1: Figures S1-S4 — Figure S1 shows titrations where the number of HMM bound actin filaments (step IV, Figure 3) is shown as a function of antigen concentration (0.5-50 nM). Figure. S2 shows a fluorescent micrograph illustrating aggregation of magnetic microparticles. Figure S3 and Figure S4 show schematic illustrations of filament fragmentation (S3) and magnetic nanoparticle aggregation (S4). [file 1477-3155-11-14-S1.doc]

**Figure S1 Number of filaments as a function of antigen (rhodamine-rIgG) concentration in two different experiments following protocol in main Fig. 3.**  Each data point corresponds to average values from measurements in three different 80 x 80 µm^2^ areas in a give flow cell. Error bars represent standard deviation between different flow cell areas.

**Aggregation**


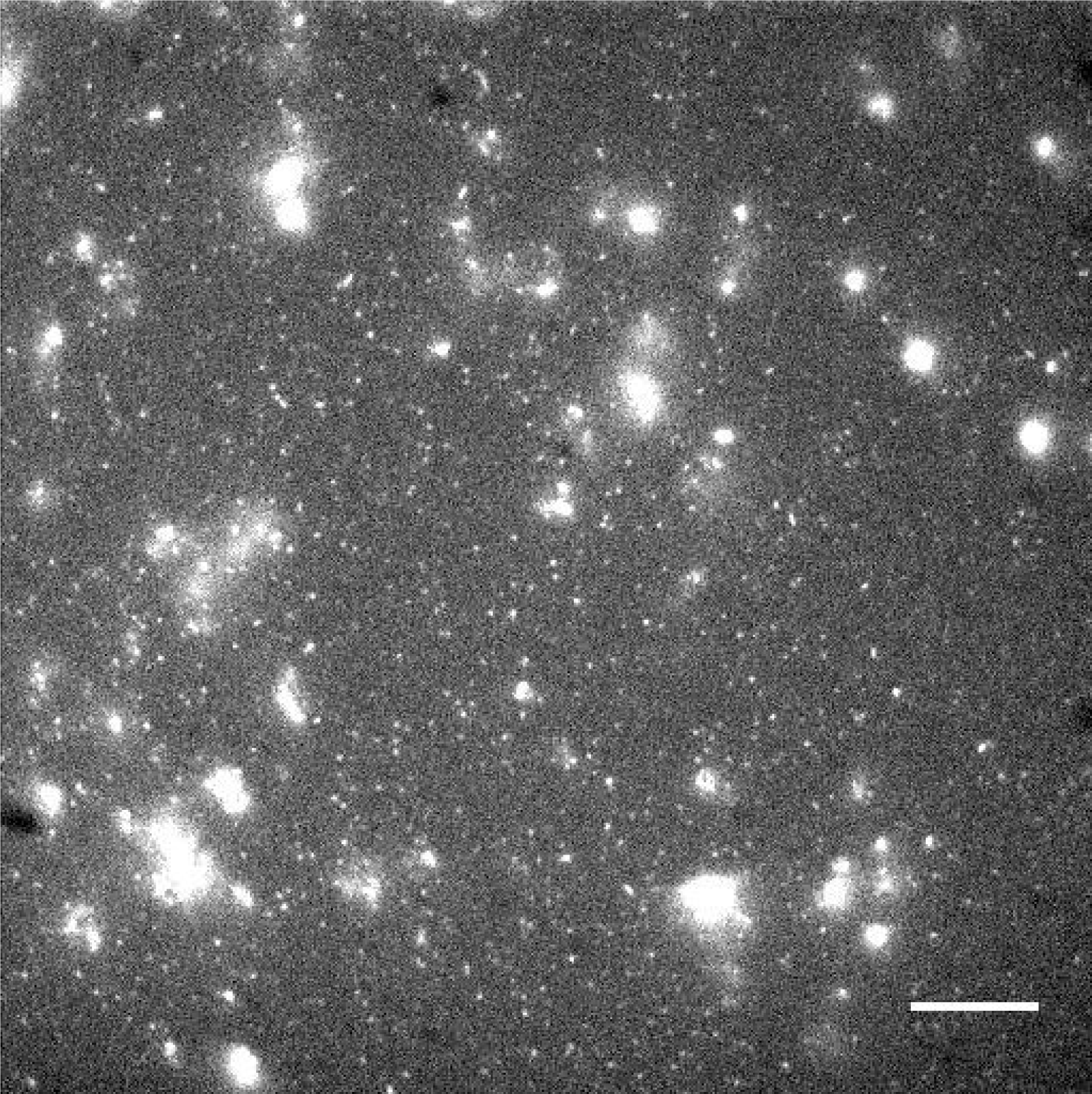


**Figure S2 Aggregation of magnetic microparticles.** Fluorescent micrograph from experiment where magnetic microparticles with antibodies, actin filaments conjugated with antibodies and the relevant antigen were mixed as described in the text**.** Here, several bright spots can be seen suggesting aggregation between actin filaments and magnetic microparticles. Scale bar 10 µm.


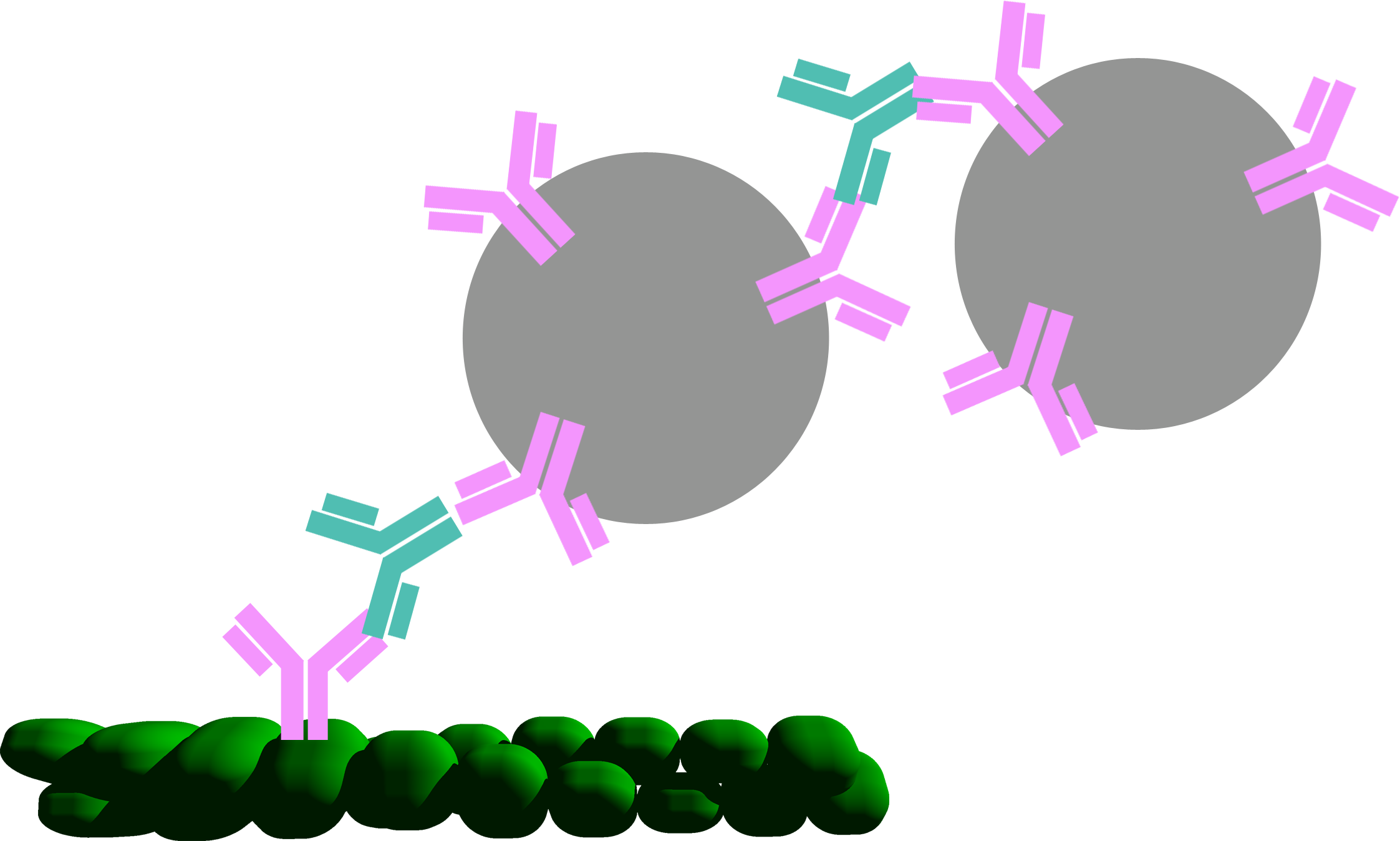


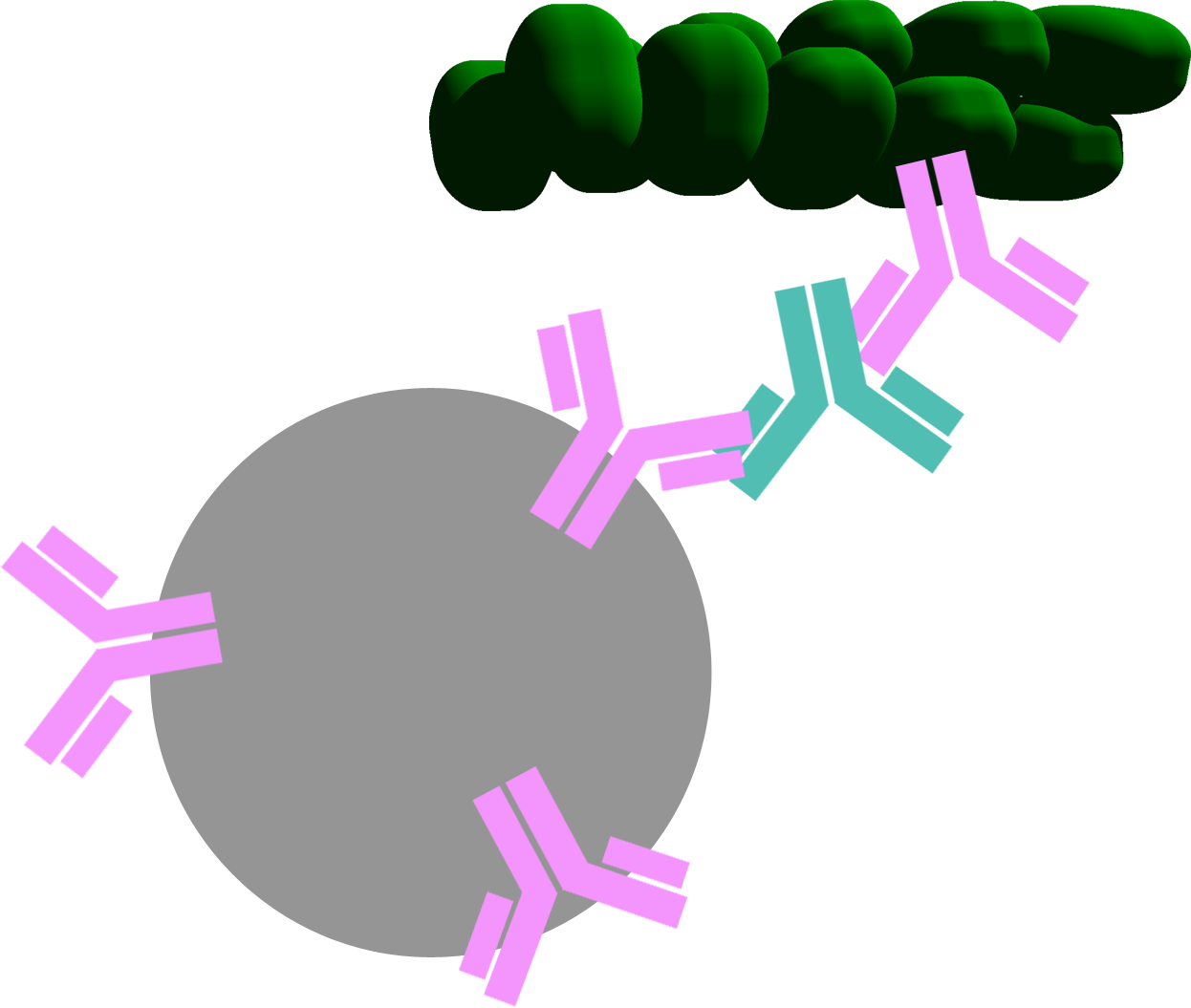


**Figure S3 Fragmentation of actin filaments due to shearing forces.** Filament (green)-antibody (pink)-antigen(blue)-antibody(pink)-magnetic particle(gray) complexes are likely to be fragmented due to shearing forces both during magnetic pelleting, resuspension and mixing. Whereas not explicitly illustrated it should be clear that such fragmentation could give rise to considerably more actin filaments than MP-antigen complexes in step IV of main Fig. 3.


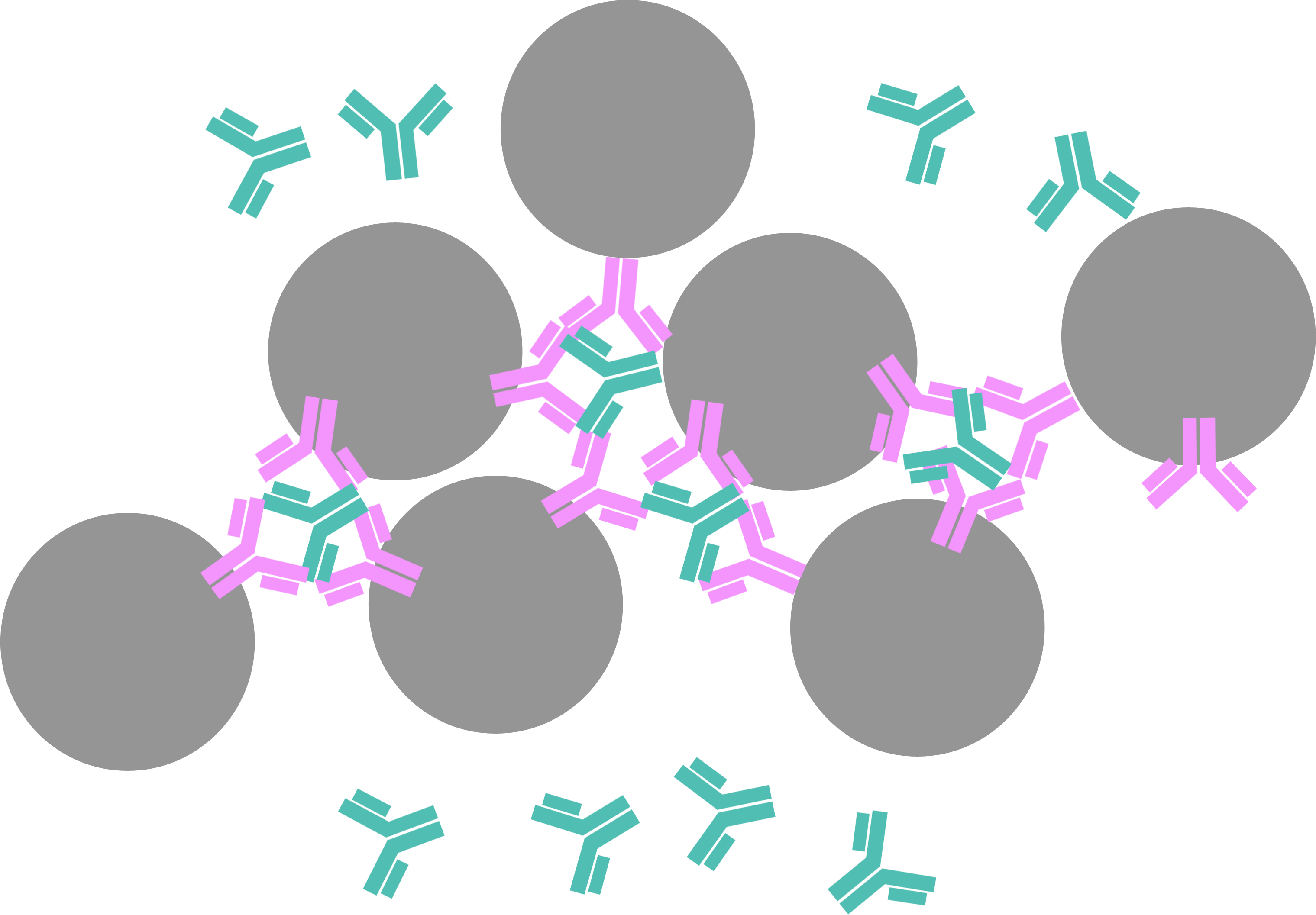


**Figure S4 Aggregation of magnetic microparticles (MPs).** Schematic illustration of MP aggregation due to the polyclonal nature of the antibodies. Conjugated antibodies (pink) on the Magnetic particles (gray) are able to bind to the same antigen (blue), forming clusters of magnetic particles with few available antibodies to bind new antigens. Figure approximately to scale with magnetic particle average size of 30 nm (manufacturer) and antibodies (IgG) approximately 5x5x15-20 nm. From microscopic observations, the aggregates are expected to be large (20-100 MPs) but are shown here as small aggregates to illustrate the principle.
